# Spatter matters – distinguishing primary (eruptive) and secondary (non-eruptive) spatter deposits

**DOI:** 10.1038/s41598-018-27065-1

**Published:** 2018-06-15

**Authors:** T. J. Jones, B. F. Houghton, E. W. Llewellin, C. E. Parcheta, L. Höltgen

**Affiliations:** 1 Department of Earth Sciences, Durham University, South Road, Durham, DH1 3LE UK; 2 Department of Geology & Geophysics, SOEST, University of Hawai’i at Mānoa, Honolulu, HI 96822 USA; 3 U.S. Geological Survey, Hawaiian Volcano Observatory, Hawaii Volcanoes National Park, HI 96718 USA; 4 Department of Earth and Environmental Sciences, LMU Munich, Theresienstr. 41, 80333 Munich, Germany

## Abstract

Spatter is a common pyroclastic product of hawaiian fountaining, which typically forms vent-proximal ramparts or cones. Based on textural characteristics and field relations of spatter from the 1969 Mauna Ulu eruption of Kīlauea, Hawai’i, three spatter types were identified: (1) Primary spatter deposited as spatter ramparts and isolated cones during the peak of episode 1; (2) Late-stage spatter comprising dense, small volume, vent proximal deposits, formed at the end of episode 1; (3) Secondary spatter preserved in isolated mounds around tectonic ground cracks that we interpret to have formed by the disruption of overlying lava. We propose that not all spatter deposits are evidence of primary magmatic fountaining. Rather, deposits can be “secondary” in nature and associated with lava drain-back, disruption, and subsequent ejection from tectonic cracks. Importantly, these secondary pyroclastic deposits are difficult to distinguish from primary eruptive features based on field relations and bulk clast vesicularity alone, allowing for the potential misinterpretation of eruption vents, on Earth and in remotely sensed planetary data, thereby misinforming hazard maps and probabilistic assessments. Here, we show that vesicle number density provides a statistically-robust metric by which to discriminate primary and secondary spatter, supporting accurate identification of eruptive vents.

## Introduction

Basaltic hawaiian fountaining can produce a range of pyroclastic products defined by the cooling efficiency within the parent fountain^1,2^. The continuous spectrum ranges from brittle basaltic scoria and spindle bombs, which have experienced substantial cooling, to hot fluidal clasts – termed spatter – that agglutinate (flatten and/or adhere)^2^ on landing because they remain predominantly above the glass transition temperature (T_g_)^3^. The degree of spatter welding depends on the clasts’ temperature upon impact and the accumulation rate. For a low cooling rate and/or high accumulation rate, spatter becomes rheomorphic, and may feed a clastogenic lava flow^1,4–8^. For a moderate cooling rate and/or low accumulation rate, the spatter forms agglutinated cones and ramparts as summarized by the classification scheme in Sumner *et al*.^2^.

The locations of pyroclastic products, such as scoria cones and spatter, observed in the field and by remote sensing are commonly used to identify the sites of previous eruptions^9–12^. Mapping of eruption vents has been performed on basaltic fissure systems, such as on Etna volcano, Italy, to produce a spatial probability map for the location of future volcanic eruptions^12^. Similarly, the identification of volcanic landforms has been used to infer volcanic activity on other planets^13,14^. However, most of this work relies on the assumption that spatter, and/or other pyroclasts, is erupted from a primary magma pathway transporting material to the surface. We note that there are three counter-examples recognized in the literature: rootless cones, which form through explosive interactions as lava propagates over a water-saturated, unconsolidated substrata^15,16^; hornitos, which form on the top of lava, by pressurized ejection of lava through gaps in the lava crust^7^; and littoral cones, which form through secondary disruption as lava enters the sea^17^. In this study, we will explore another enigmatic type of secondary spatter pyroclastic deposit that, to our knowledge, has never been described before.

The quantification and analysis of vesicle size distributions (VSD) and vesicle number densities (VND) in eruptive products is commonly used to constrain ascent and decompression processes^18–20^, conduit dynamics^21^, and bubble nucleation and growth^22–25^. Importantly for this study, the vesicle size or volume distribution preserved within a pyroclast is not simply a function of nucleation rate or volatile concentration, but instead is modified through time by expansion, coalescence, and collapse processes^22,26,27^. Hence, the quantitative textural analysis of the vesicle population can provide information on the timing of magma fragmentation and the extent of shallow conduit recycling (i.e. upper 10’s of m) and surface flow^21,28^. Previously, vesicularity studies of pyroclasts from hawaiian fountains have been largely confined to high fountaining episodes^19,20,27,29,30^, with only Parcheta *et al*.^31^ analysing low-fountaining activity (defined as a fountain with height <100 m)^32^. In this paper we expand the quantification of vesicle textures for low fountaining hawaiian eruptions and set out a framework for distinguishing primary from secondary spatter.

## Field evidence for a new type of spatter

Field investigation of the episode 1 deposits of Mauna Ulu identified spatter that is not associated with a primary volcanic vent. In this section we briefly review the field context that underpins this inference.

### The 1969-74 Mauna Ulu eruption

**Figure 1:**
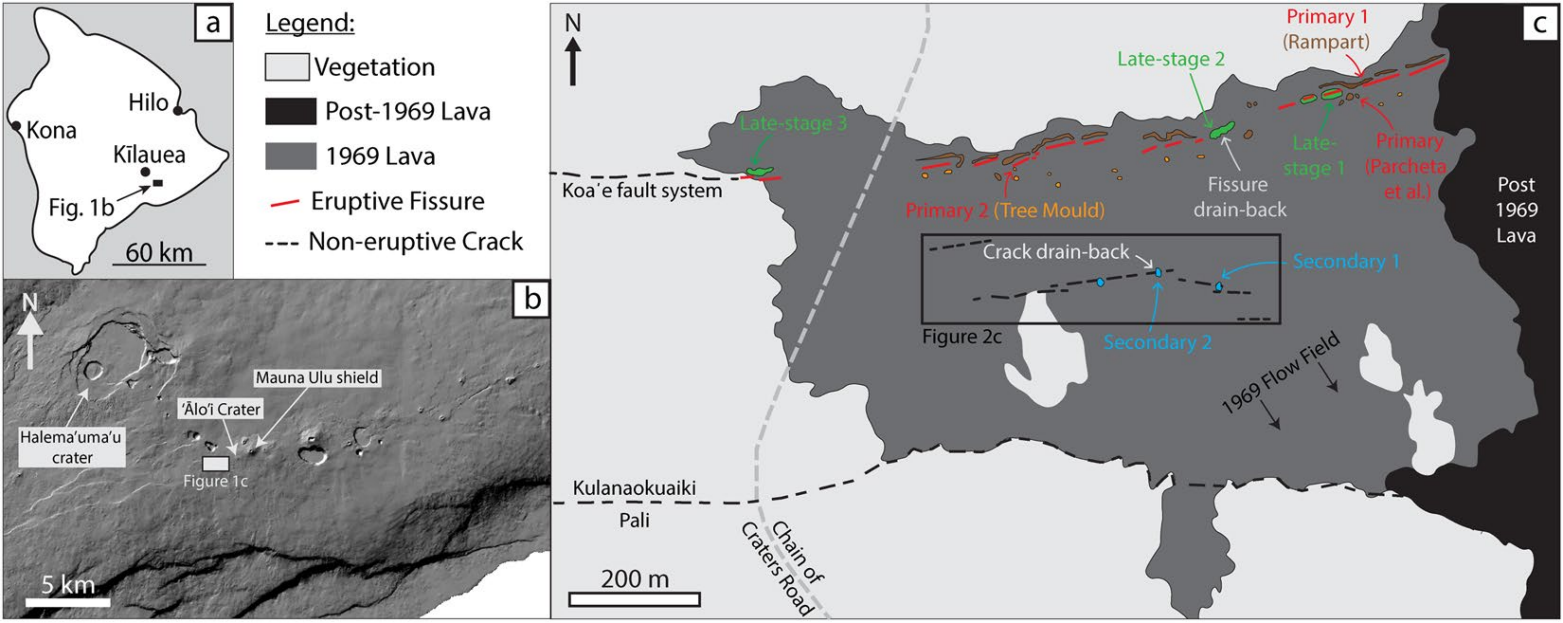
(**a**) Overview map of the Island of Hawai’i with the location of our field area marked. (**b**) The location of Mauna Ulu with reference to other landforms on the southern flank of Kīlauea. Modified from Jones *et al*.^35^ and reproduced under the Creative Commons Attribution 4.0 International License (http://creativecommons.org/licenses/by/4.0/). (**c**) Interpretative map of the Mauna Ulu 1969 episode 1 fissure vents and associated products. Colour-coded arrows indicate locations where spatter and lava samples were taken. The central black box marks the field of view in Fig. 2c. Numbering only distinguishes different sampling locations and has no temporal meaning. Note that the modern Chain of Craters Road labelled in this figure is in the same location as the old ‘Āinahou Road described by Swanson *et al*.^33^.

Mauna Ulu is located on the East Rift Zone (ERZ) of Kīlauea volcano, Hawai’i (Fig. 1). On May 24^th^, 1969, episode 1 of the 1969–74 eruption initiated with an earthquake swarm. At 04:45 Hawaiian Standard Time (HST) low fountaining began from a newly formed fissure system, east of ‘Ālo’i Crater^33^. The fissure system rapidly propagated 1 km eastwards and 3 km westwards, crossing the old, now abandoned, Chain of Craters Road by 05:00 HST and crossing ‘Āinahou Road (which has become the current Chain of Craters Road; Fig. 1) at 08:30 HST^33^. Steady fountaining and lava ponding around the fissure occurred until 1200–1300 HST^34^ and fed a southward-advancing pāhoehoe field. The steady fountaining was followed by a period of waning fountaining and lava drain-back ending at 22:00 HST^34,35^. This was the end of episode 1 fountaining in our field area (Fig. 1), but subsequent episodes occurred to the east, where weak episode 1 fountaining continued until ~15:00 HST on May 25. All spatter described here is related to the episode 1 activity, however for a complete narrative of the 1969–74 Mauna Ulu eruption, readers are directed to Swanson *et al*.^33^ and Tilling *et al*.^36^.

### Tectonic ground cracks: field evidence and eyewitness accounts

In addition to the fissure containing the eruptive vents at Mauna Ulu, there are several large tectonic ground cracks approximately 100–200 m to the south, oriented roughly parallel to the fissure (marked in a black box in Fig. 1). Based on their location and orientation, the cracks are likely associated with the ongoing tensional opening of the ERZ caused by the seaward movement of Kīlauea’s southern flank^37,38^. Swanson *et al*.^33^ reported that ground cracks in this region opened during the morning of April 9^th^, 1970 *“accompanied by emission of fume”* and propagated westwards to reach the current Chain of Craters Road by 10:00 HST April 10^th^. Importantly for this study, Swanson *et al*.^33^ describe that these cracks did not act as eruption sites. They also document that these cracks widened through time, growing to widths of ~1 m. However, following the field evidence of Parcheta *et al*.^34^ we now know that this 1970 event was, probably a reactivation of older tectonic cracks and/or creation of new ground cracks.

**Figure 2:**
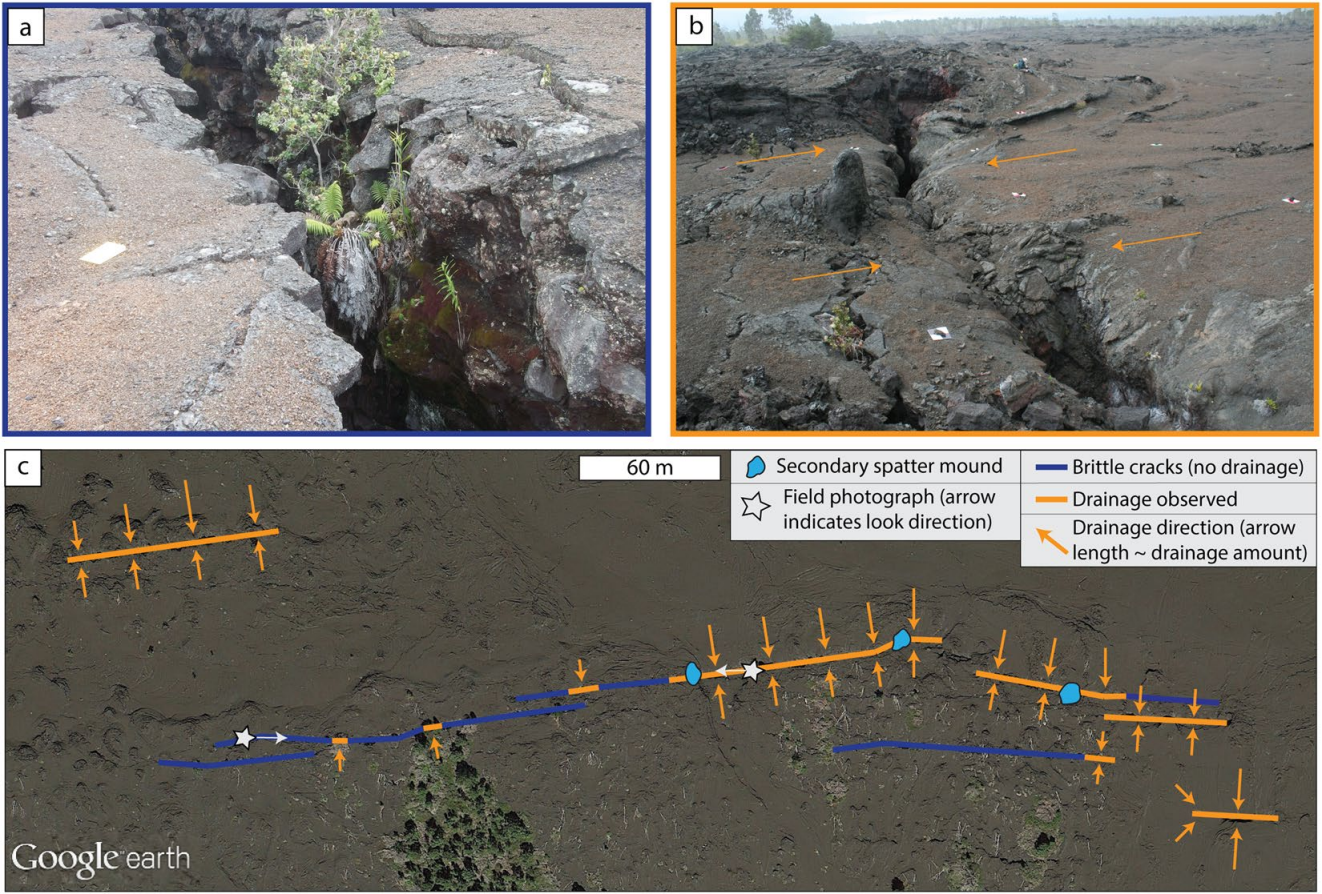
Field photographs and aerial images of the ground cracks. (**a**) Complementary jigsaw-fit ground cracks cross-cutting the 1969 surface lava (and older lavas at depth). There is no evidence here that the surface 1969 lava was mobile when the crack opened. For scale, the white rectangle in the photograph measures 24 cm by 13 cm. (**b**) Prominent southward-draining lava channel cut by a ground crack. Lava can be observed draping down into the crack. Ground squares measure 30.5 cm by 30.5 cm. (**c**) Annotated Google Earth image showing regions of the southern ground cracks where either simple brittle ground cracking is observed (marked in blue) or where drainage of the surface lava down into the cracks is observed (marked in orange). The length of the orange arrows indicates the relative amount and direction of drainage. The photograph locations and look directions are indicated by the white stars.

To establish a relative timing of crack opening and emplacement of the 1969 lava, we mapped the morphological characteristics of the ground cracks. We observed two distinct features: firstly, piercing points, which are simple saw-tooth edges where complementary ‘jigsaw-fit’ shapes can be observed at either side of the crack (Fig. 2a); secondly, a mantle of draping lava, indicating drainage of 1969 surface lava into the crack (Fig. 2b). The well-developed 1969 lava channels are cross cut by the ground cracks that show crack-drainage (Fig. 2b) and in some cases the cracks have small (<3 m) vertical offsets that displace the lava high-stand surface. Thus, we can interpret the relative timing of crack opening: cracks associated with simple saw-tooth morphology formed after the 1969 lava had cooled, most likely in April of 1970 (blue; Fig. 2c); whereas segments with lava drainage drapery opened/widened during the waning stages or immediately after the May 1969 eruption (orange; Fig. 2c).

### Episode 1 spatter deposits

**Figure 3:**
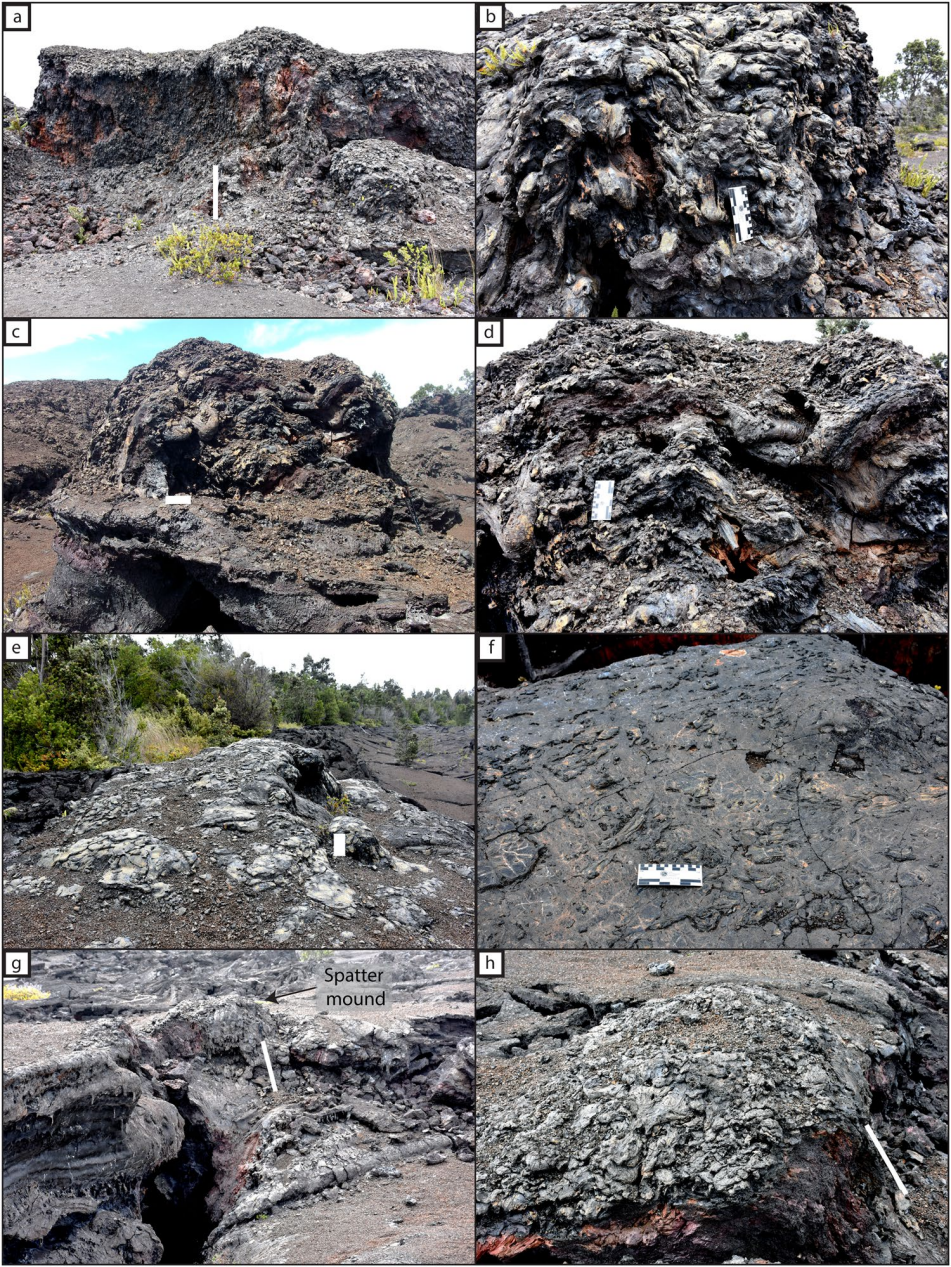
Field photographs of the three different spatter types described in this study: primary (**a–d**); late-stage (**e**,**f**) and secondary (**g,h**). (**a**) A spatter rampart that formed on the northern side of the 1969 Mauna Ulu eruptive fissure; (**b**) agglutinated rampart interior with many clasts showing post depositional flow; (**c**) primary spatter located on top of a tree mould south of the eruptive fissure; (**d**) interior of tree mould spatter mound, where clasts are less welded compared to those in the northern rampart deposits; (**e**) green/blue vent proximal late-stage spatter forming a thin deposit on top of the drain-back lava; (**f**) typical late-stage spatter clasts showing a wide range of fluidal shapes; (**g**) a secondary spatter mound formed over a ground crack to the south and (**h**) a close up view of a secondary spatter mound. Scales (marked in white if unclear) measure 16 cm for all images except in a, g and h where the scale is 1.2 m.

Episode 1 of the Mauna Ulu eruption produced three distinctly different types of spatter deposit. The main primary deposit^34^, is to the north and intermittently to the south of the eruptive fissure as a discontinuous series of asymmetrical spatter ramparts (Fig. 3a). The ramparts can be found along 80% of the fissure length on the northern side, they vary considerably in height from 0.7 m to 7.1 m, and are typically set back by ~10 m from the eruptive vent^34^. Clasts within the ramparts are highly agglutinated (especially towards the deposit base) and elongate (Fig. 3b). South of the eruptive fissure, isolated spatter mounds can be found on the top of tree moulds^34^. Clasts preserved on the tree moulds show less agglutination and are more fluidal in shape relative to those observed in the rampart interior (Fig. 3d). Both the spatter found within the ramparts and on top of tree moulds are the products of episode 1 fountaining deposited proximally to the vent^33^. Therefore, in this work, this type of spatter is termed ‘primary spatter’.

A second type of spatter deposit is found overlying ‘drain-back’ lava at the primary fissure. Flow directions quantified in previous studies show that, during the waning stages of episode 1, some of the lava drained back into the fissure vents^33–35^. In some locations, and only within ~5 m of the eruptive vents, clasts of spatter are found on top of the back-draining 1969 lava carapace (Fig. 3e). This spatter does not form mounds or ramparts because the deposit is rarely greater than a single clast thick. The clasts are fluidal in morphology, varying from highly elongate strands and droplets to more circular discoidal shapes and have a dark green/blue surface colour (Fig. 3f). It is likely that the larger clasts broke up upon impact forming adjacent smaller droplets^2^. The field relations indicate that this spatter was emplaced following lava drain-back into the fissure vent, hence we term this ‘late-stage spatter’.

A third, rare type of spatter deposit is observed associated with the southern tectonic ground cracks (Fig. 2). As previously described, these fissures are known not to have erupted any magma during the eruption, yet prominent spatter mounds (Fig. 3g) occur at three locations along these cracks (Fig. 1). Without eye-witness accounts, it would be extremely difficult to distinguish portions of these ground cracks from the main 1969 eruptive vents to the north. The spatter is concentrated very close (<1 m) to the cracks (Fig. 3g), although rare individual single clasts can be found up to ~6 m on either side of the crack. The discrete mounds are only found on relative topographic highs and/or at an en echelon step. The clasts themselves (Fig. 3h), are highly variable in physical appearance; many isolated clasts (i.e. those not forming mounds) are thin discs and sheets whereas clasts forming mounds are slightly rheomorphic. Many clasts are cracked, and have a green/blue outer colour, similar to the late-stage spatter. We term this type of spatter on the non-eruptive cracks as ‘secondary spatter’.

## Methods

### Field sampling

Around 100 clasts of each sample type were collected during March 2015 and May 2017. During sampling, clasts were chosen at random from a small area and, where the deposits were thick, samples were limited to single stratigraphic horizons. Figure 1 marks the exact sample locations. The primary spatter was collected from the interior of one of the northern ramparts, towards the eastern end of the eruptive fissure, and from the top of a tree mould located 4.2 m south of the vent. The spatter from the rampart and tree mould collectively provide the primary spatter dataset. The late-stage spatter was sampled in three locations along the eruptive fissure: at the western end of the eruptive fissure, where it joins the Koa‘e fault system, in the low eastern section of the episode 1 fissure where the vent is completely covered in lava, and at a flared, circular vent segment, towards the eastern end of the eruptive fissure as currently exposed. The secondary spatter was collected from two prominent mounds (Secondary spatter 1 and 2; Fig. 1) adjacent to the southern ground cracks. For bulk vesicularity comparisons, samples of lava crust that were obviously *in-situ* were broken up and sub-samples collected at random. This was done at two localities: directly above the, now covered, eruptive fissure labelled as “Fissure drain-back” and adjacent to the ground cracks labelled as “Crack drain-back” (Fig. 1).

### Bulk vesicularity

A minimum of 95 clasts from each sample type (spatter and lava) were measured for bulk density using the Archimedes techniques outlined in Houghton and Wilson, (1989)^39^. The bulk vesicularity was calculated using a dense rock equivalent density (2873 ± 5 kg m^−3^) measured independently by He pycnometry. From five repeat measurements on the same spatter clast we report a maximum absolute uncertainty associated with each clast bulk vesicularity determination of ±1% vesicularity. The variation in vesicularity among different spatter types is considered later.

### Quantitative vesicle analysis

**Figure 4:**
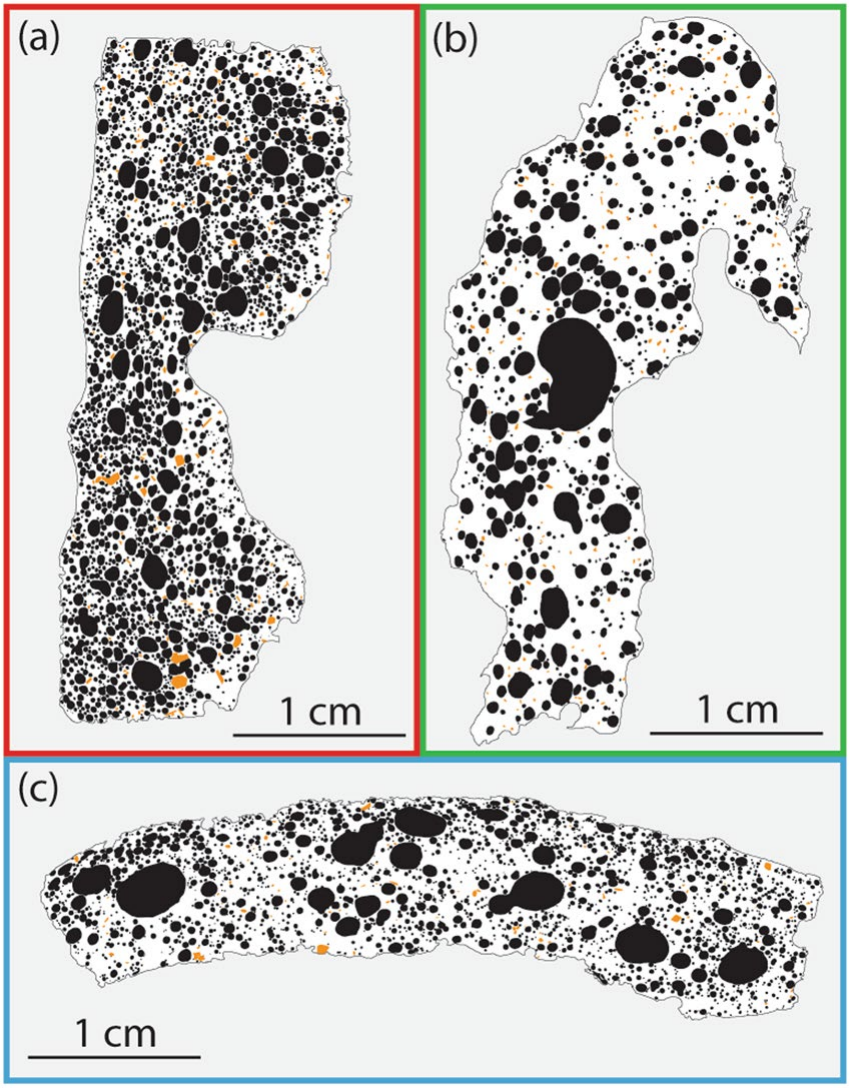
A representative binary thin section image for: (**a**) primary spatter; (**b**) late-stage spatter and (**c**) secondary spatter. Large vesicles and phenocrysts are shaded black and orange respectively.

From the samples measured for bulk density, 14 were selected for quantitative vesicle analysis; samples were chosen to cover a range of spatter types and bulk vesicularity. If a clast had a quenched rim and a micro-crystalline core, the two parts were separated and only the quenched rim was analysed as the bubbles in the core have been modified by crystallisation and inflation after deposition. The quenched rims, and therefore all our data, represent textures immediately following clast formation. A nested image analysis approach^22^ was adopted to accurately capture all bubble sizes using: (1) full thin section scans taken at 4800 dpi on a flatbed scanner and, (2) large back-scattered electron (BSE) image grids taken at 130× magnification on a Hitachi SU-70 scanning electron microscope (SEM). The SEM operating conditions were: a 15 kV accelerating voltage, a 45 μA beam current, a 15 mm sample working distance, and a 32 μs dwell time during image acquisition. All the images were then manually traced using Inkscape or Adobe Illustrator to create binary masks of vesicles and phenocrysts (Fig. 4). We note that, during tracing, vesicles were not heavily de-coalesced; only undeformed vesicles with thin (~≤50 μm) joining septa were de-coalesced. Quantification of the binary masks was performed with ImageJ software to obtain vesicle areas and ultimately the 2D areal vesicle number density (N_A_). N_A_ was then corrected for phenocrysts and converted to a volumetric (3D) vesicle number density (N_V_) following the method of Proussevitch and Sahagian (1998)^40^. In samples where only the quenched rim was analyzed (denoted by a “q” in the sample names) we normalized to the image vesicularity, rather than the bulk vesicularity, after Stovall *et al*.^29^. Lastly, N_V_ was referenced to the melt volume to account for the volume occupied by the vesicles themselves^25^. This forms the final metric, N_Vm_ that we use throughout this study.

### Data availability

The datasets generated during and/or analysed during the current study are available from the corresponding author upon request.

## Results

### Bulk clast vesicularity

**Figure 5:**
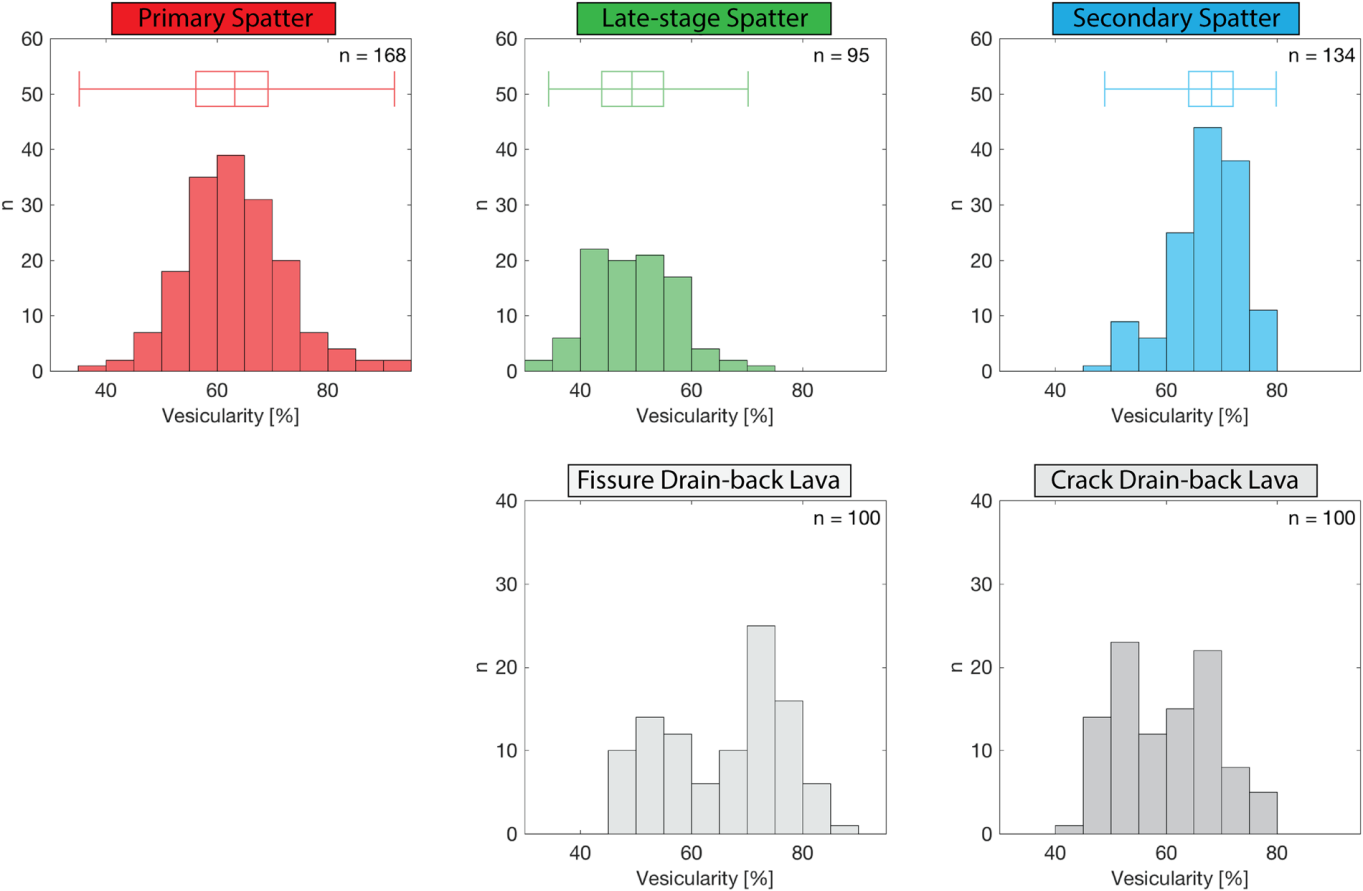
Bulk vesicularity histograms for all spatter types, the drain-back lava sampled close to the fissure vent, and the drain-back lava along the southern ground cracks. The number of samples (n) measured is reported for each dataset. The box and whisker plots display the data range (whiskers) and the first quartile, median and third quartile (box). Box and whisker plots were not calculated for the lavas, as these data are clearly bimodal.

Figure 5 shows the bulk vesicularity histograms for the primary (red), late-stage (green) and secondary (blue) spatter with median bulk vesicularity values of 63%, 49% and 68% respectively. The primary spatter displays a normal, unimodal distribution. The late-stage spatter is also unimodal but has a broader peak. However, the secondary spatter shows a slight bimodality with modes at 50–55% and 65–70%. As indicated by the box-plots in Fig. 5, the secondary spatter samples are tightly distributed, with the smallest inter-quartile range of all the sample types.

Samples of lava preserved proximally to the eruptive fissure also show a bimodal distribution with modes at 50–55% and 70–75% vesicularity. These two modes reflect vesicle poor and vesicle rich domains in the lava respectively. Lava samples close to the southern ground cracks again show a bimodal distribution with similar subpopulation modes at 50–55% and 65–70% vesicularity. We note that the relative proportion of these subpopulations changes between the two lava types; the crack drain-back lava is relatively enriched in the denser subpopulation and depleted in the more vesicular subpopulation. Furthermore, although most of the lava is spatter-fed, it is extremely rare to find any remaining textural evidence of a rheomorphic origin.

### Qualitative textural observations

The three spatter types are texturally distinct on visual inspection. The primary spatter contains abundant small vesicles that are mainly spherical, although, some vesicles are elongated, especially those close to a clast margin (Fig. 4a). In contrast, the late-stage spatter has much coarser vesicles and very few small vesicles. The vesicles themselves are spherical, except where coalescence is evident (Fig. 4b). Microlites are common in the late-stage groundmass. The secondary spatter vesicle population qualitatively lies between the late-stage and primary spatter; it has a large number of small, spherical vesicles but also some larger vesicles and coalescence textures (Fig. 4c). No systematic changes in phenocryst content are observed among sample types; all samples have plagioclase and olivine phenocryst abundance ≤2.5%.

### Quantitative textural observations

#### Vesicle number densities

The melt-referenced vesicle number densities (N_Vm_) range from 1.42 × 10^4^ to 2.50 × 10^6^ vesicles per cm^3^ across all the sample types (Table S1). Primary spatter has the highest N_Vm_ with a mean of 1.67 × 10^6^ cm^−3^. In contrast, the late-stage spatter has the lowest N_Vm_ (3.87 × 10^5^ cm^−3^) and the largest range in N_Vm_ with values varying from 1.42 × 10^4^ to 7.54 × 10^5^ cm^−3^. Secondary spatter has an intermediate N_Vm_ (mean: 5.77 × 10^5^ cm^−3^). The range of vesicle number densities for secondary spatter is also very small compared to the other sample types (Table S1).

#### Vesicle size distributions

**Figure 6:**
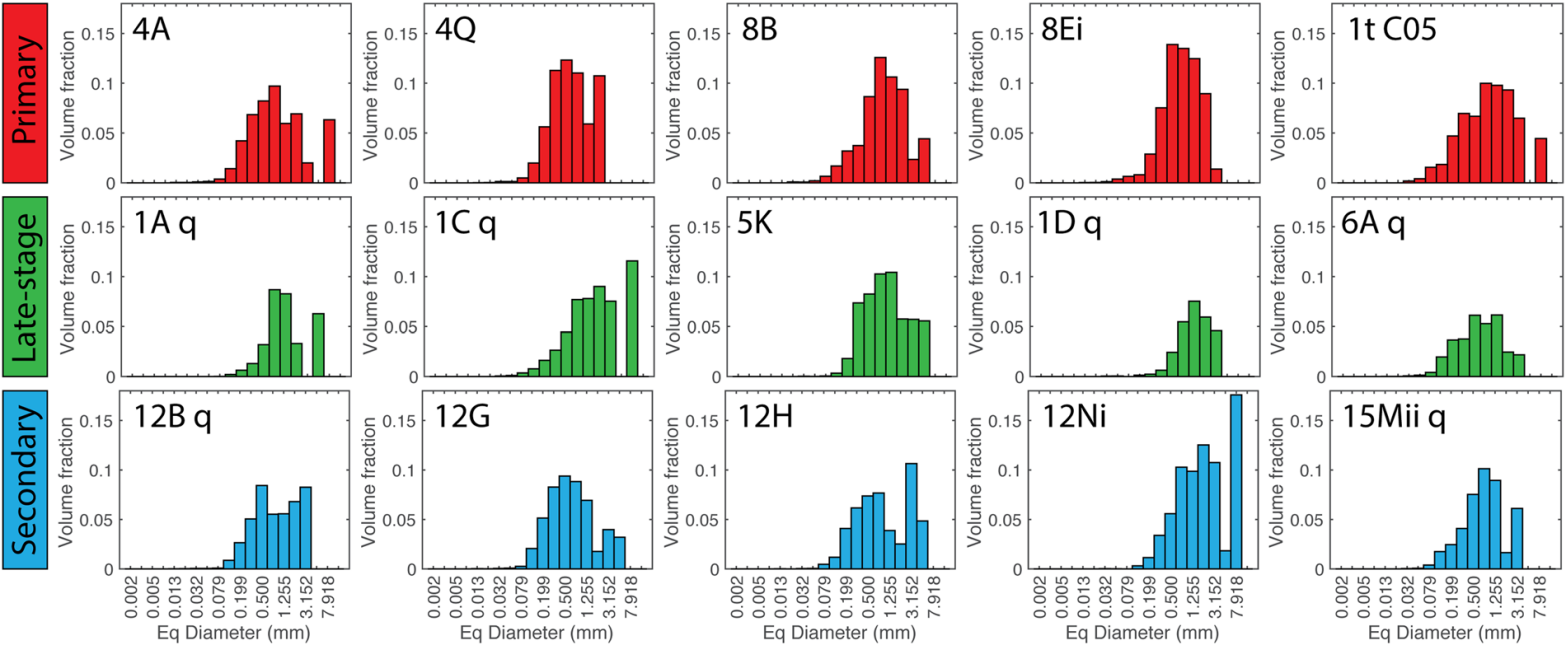
Vesicle size distribution histograms for primary (red), late-stage (green) and secondary (blue) spatter. The equivalent (Eq) diameter is calculated from the traced vesicle area, assuming a circular cross-section.

The vesicle size distributions (VSD), expressed as a volume fraction, also vary according to spatter type. Figure 6 shows VSD histograms binned for equivalent spherical diameter. Primary spatter (red; Fig. 6) displays a variably unimodal, near normal distribution with a relatively high volume fraction; a consequence of the high bulk vesicularity of primary spatter. A small secondary mode at 3 to 8 mm in some samples is due to coalescence of the largest vesicles. The late-stage spatter (green; Fig. 6) has a lower volume fraction (due to its lower bulk vesicularity), distributions that are slightly negatively skewed, and are mainly unimodal with the exceptions of samples 1C q and 1A q that have an additional coalescence-driven coarse mode at 8 mm and 5 mm equivalent diameter bins respectively. The secondary spatter volume fractions are comparable to the primary spatter, as expected from their similar bulk density (Fig. 5), however, the size distributions are distinctly different. The secondary spatter (blue; Fig. 6) VSD histograms are broad and generally bimodal with a fine mode at 0.5 mm and a coarse mode at 3.15 mm. An outlier, 12Ni, still shows a bimodal distribution but has fine and coarse modes at 1.98 mm and 7.92 mm respectively.

**Figure 7:**
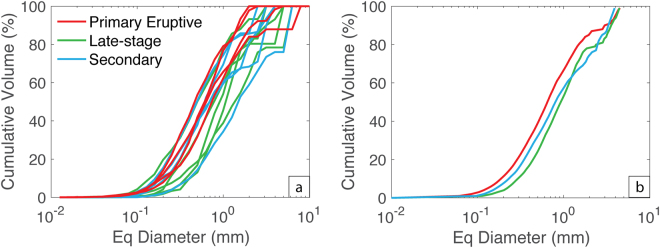
Cumulative vesicle volume % curves colour coded for sample type. (**a**) All samples, where each line represents an individual clast and (**b**) mean cumulative curves produced by calculating the average equivalent diameter for each percent from 0 to 100%.

Figure 7a presents all VSD data in terms of cumulative volume percent. To aid comparison among spatter types we calculate the mean average cumulative distribution curves for each sample type (Fig. 7b); specifically, we calculated the mean equivalent diameter for each cumulative volume from 1 to 100%. This plot clearly shows the contrast between the three spatter types both in terms of the size of the dominant subpopulation and the relative significance of a coarse, coalescence-related subpopulation. The primary spatter has the finest VSD with a median (50%) equivalent diameter of 0.65 mm. In comparison, both the secondary and late-stage spatter have coarsened distributions with median diameters of 0.80 and 0.93 mm respectively. Also worthy of note is that the late-stage spatter is extremely depleted in small vesicles but shares a similar secondary mode to the secondary spatter at high (>80%) cumulative volume fractions.

## Discussion

The production of spatter is most commonly attributed to primary magmatic fragmentation, where magma is disrupted forming hot, incandescent pyroclasts that are entrained by a rising gas jet^41^. This eruption style is typical of hawaiian fountaining and produces spatter cones/mounds, ramparts and/or clastogenic lava flows^2,6,8^, although it is not limited to mafic volcanism – similar deposits are associated with felsic magmas^42,43^. However, in all these cases spatter mounds and ramparts are located proximally to the vent system, and the presence of spatter may be taken as indicative of proximity to an eruptive vent. Spatter deposits have been found to occur along tectonic ground cracks, but only where those cracks have been occupied as magma ascent pathways during eruption. For example, at Gjástykki, Iceland, magma rose along pre-existing ground cracks, leading to the formation of spatter cones^44^.

In this work, we have shown that spatter mounds can also be associated with non-eruptive ground cracks into which lava drained. The clear spatial clustering of secondary spatter along the cracks removes the possibility that simple rafting of primary spatter on top of lava flows formed the deposits. Based on field observations alone, and in the absence of microtextural analysis of the pyroclasts, certain regions of these ground cracks could easily be mistaken for primary eruptive vents. Secondary spatter (as spatter mounds) associated with the ground cracks is difficult to distinguish from primary spatter in the field since both deposits have comparably high bulk vesicularity, are located along fissures/cracks and lie on top of co-existing lavas. Therefore, field observations alone are not enough to identify the difference between primary and secondary spatter, and a more detailed micro-textural analysis is required.

### Micro-textural interpretations

**Figure 8:**
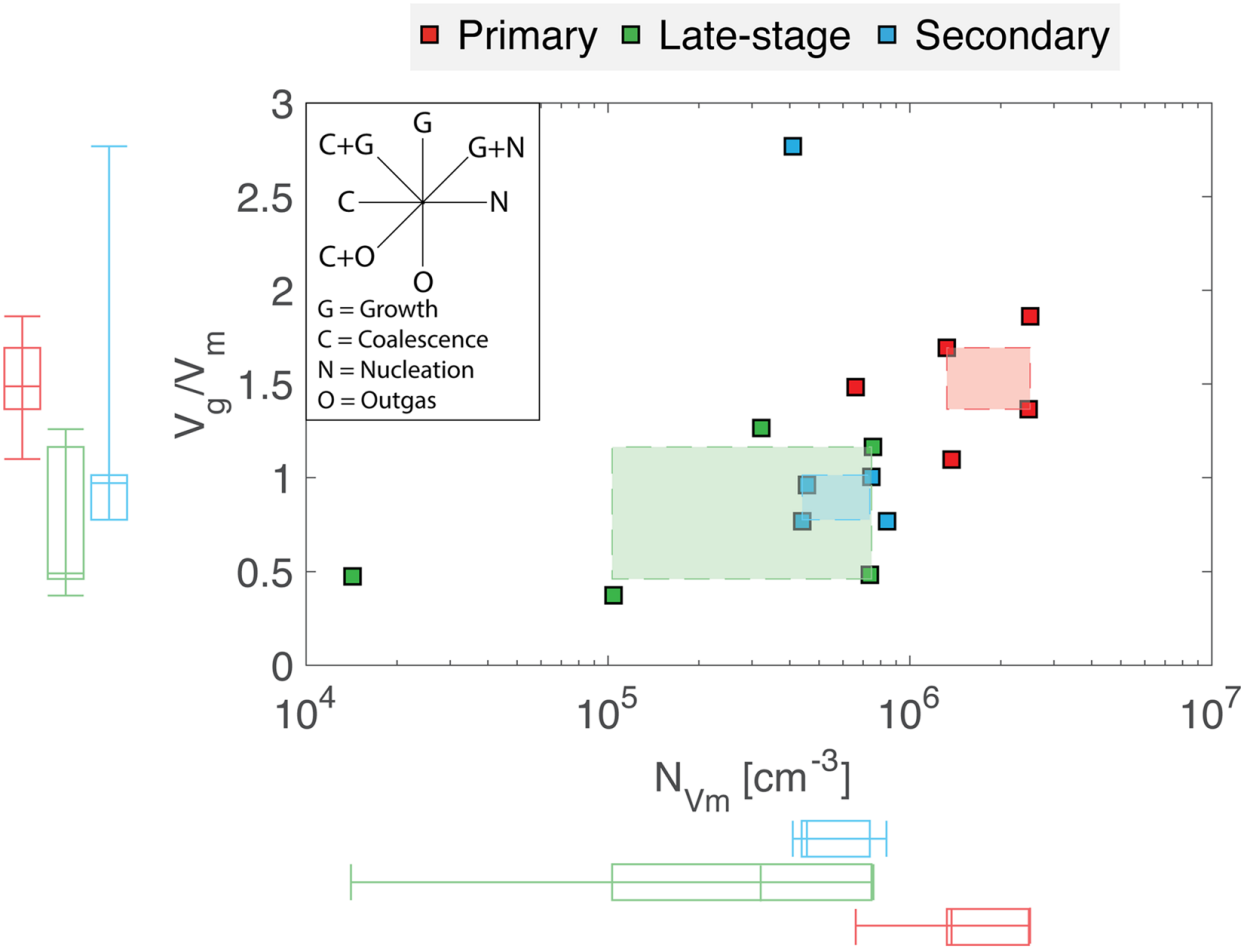
Volume ratio of gas (V_g_) to melt (V_m_) plotted against the melt-referenced vesicle number density (N_Vm_) for all spatter types. The box and whisker plots display the data range (whiskers) and the first quartile, median and third quartile, collectively forming the inter-quartile range (IQR; box). Shaded fields represent the overlapping IQR of both V_g_/V_m_ and N_Vm_. Primary spatter can be distinguished from the secondary and late-stage spatter based on vesicle number density (N_Vm_).

In hawaiian pyroclasts, vesicle number densities and size distributions can reveal information on bubble nucleation, growth, coalescence, the timescales of magma ascent and the influence of post-fragmentation expansion, if present, within the thermally insulated fountain interior^20,22,27,29,30,45,46^. However, the micro-textural study of mafic melts provides challenges. Their high temperature and low viscosity promote post-eruption bubble expansion and coalescence, which can overprint original shallow conduit textures^31^. In our case, however, this is advantageous; the textures we wish to contrast are those at final deposition and not necessarily the vesicle textures at primary fragmentation. To aid comparison between the spatter types, we plot (Fig. 8) the melt-referenced vesicle densities (N_Vm_) as a function of the ratio of the volume of gas *V*_*g*_ to volume of melt *V*_*m*_ after Stovall *et al*.^29^. Specifically, *V*_*g*_/*V*_*m*_ = *ϕ*_v_(1 + *ϕ*_*p*_)/(1 − *ϕ*_*v*_), where *ϕ*_*v*_ is the volume fraction of vesicles and *ϕ*_*p*_ is the modal proportion of phenocrysts^47^. On this plot (Fig. 8; top-left sub panel), different trends denote different physical processes; for example, a decrease in the gas to melt volume ratio without changing the vesicle number density would indicate pure outgassing (the loss of gas from the system).

The primary spatter has the highest vesicle number densities and gas to melt volume ratios recorded in this study. The VSD of primary spatter sampled is unimodal reflecting one period of nucleation and growth followed by minor coalescence. However, if a range of primary pyroclasts were to be sampled systematically from fountains of different height and/or from different positions within a fountain, or for a wide range of particle sizes, we would expect to see a range of vesicle textures resulting from different degrees of overprinting by coalescence and/or expansion. This has been previously observed at Mauna Ulu^31^ and Kīlauea Iki^27,29^ where increased coalescence was interpreted to be a result of longer residence time in the hot interior of the fountain (10’s of seconds available for coalescence).

The late-stage spatter has much lower vesicle number densities and slightly lower gas to melt volume ratios compared to the primary spatter (Fig. 8; Table S1). The change from the primary spatter to the late-stage spatter indicates an evolution involving both outgassing (escape of gas) and coalescence of new bubbles after nucleation had ceased (Fig. 8). Furthermore, this is supported by the VSDs (Fig. 7) that show a coarsening and increased bimodality resulting from the reduced abundance of small vesicles as early, nucleated bubbles had expanded and coalesced. Additionally, the bulk vesicularity of the late-stage spatter is on average 13.5% lower than the primary spatter (Fig. 5), supporting the interpretation that the magma producing these late-stage clasts was more outgassed.

The micro-textural data from the secondary spatter reveals a bimodal VSD (Fig. 6) and a reduced vesicle number density relative to the primary spatter (Fig. 8) interpreted to result from bubble coalescence. The secondary VSD is coarser than the primary spatter but finer than the late-stage spatter at equivalent vesicle diameters <63 μm but becomes coarser or comparable to the late-stage spatter at larger equivalent vesicle diameters. This suggests that the secondary spatter underwent less coalescence than the late-stage spatter and was able to retain (not outgas) more of its larger bubbles.

We have shown the difficulty in identifying spatter types based on bulk density and field relationships alone. Now, the hypothesis that the three spatter types (primary, late-stage and secondary) can be separated based upon their vesicle number density (N_Vm_) is tested quantitatively using a two-way analysis of variance (ANOVA). The ANOVA test results are shown in Table S2. The null hypothesis (the two sample types are drawn from the same distribution) can be rejected at the 0.05% level (P-values < 0.05) for Primary – Late-stage and Secondary – Primary comparison. This means that, based on vesicle number density (N_Vm_), primary spatter can be distinguished from both late-stage and secondary spatter. However, secondary and late-stage cannot be distinguished using this approach. An ANOVA test was also performed using V_g_/V_m_, but this produced less statistically significant separations between sample types. Therefore, N_Vm_ is the best metric to distinguish primary spatter from the other types.

### Spatter formation re-visited

Spatter is commonly described in association with (primary) magmatic fountaining driven by the expansion and exsolution of volatiles in the upper conduit^2,41^. Here, we have shown that some spatter deposits can form along ground cracks that did not act as pathways for ascending magma. This novel finding indicates a need for a more complete description of the formation conditions of the different spatter types. The formation of primary spatter has already been covered in great detail by many other studies^1–3,48^. In the case of Mauna Ulu, the primary spatter deposits were formed during episode 1 of the 1969 eruption by sustained weak fountaining reaching maximum heights of 50 m^33,34^. Primary spatter falling proximally (<20 m) downslope, downwind of the vent became incorporated into rheomorphic lava flows, whereas material falling >20 m downslope was rafted away on top of these lava flows^34^. Primary spatter was preserved locally on top of some tree moulds, which acted as high points, and local barriers to flow (Figs 1 and 3c). To the north (upwind and upslope) a near-continuous spatter rampart was formed (Figs 1 and 3a). For a complete description of primary spatter formation during episode one of the 1969 Mauna Ulu eruption, readers are referred to Parcheta *et al*.^31,34^.

**Figure 9:**
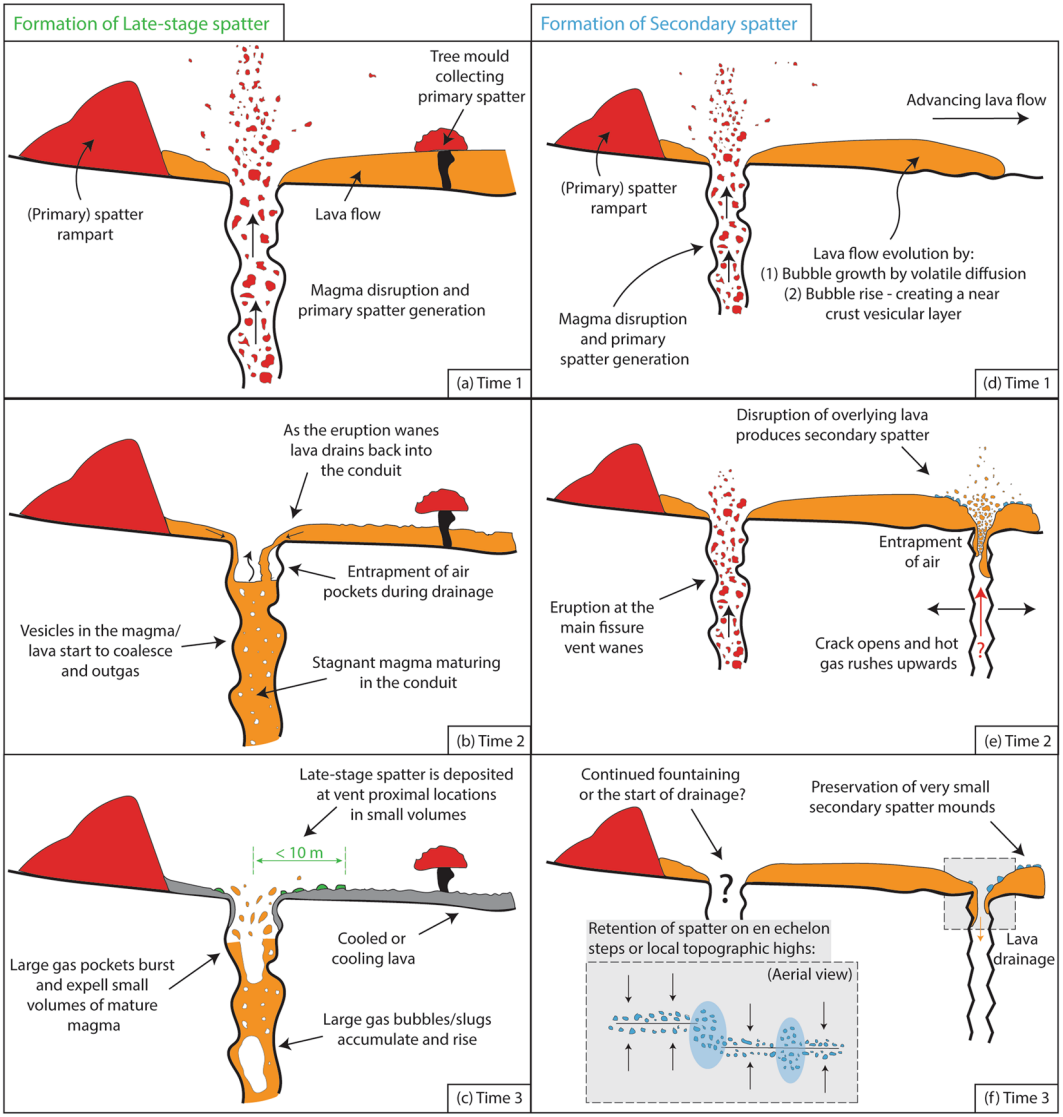
Schematic of the formation of the spatter types described in this study. Diagrams are not to scale. The view is to the east along strike of the Mauna Ulu fissure.

As the eruption and associated primary fountaining started to wane, vent-proximal ponded lava began to drain back down into the conduit locally^34,35^. Over time, the draining material and magma left stalled in the conduit matured by bubble coalescence and outgassing, causing the bulk magma vesicularity, the vesicle number density, and the gas to melt volume ratio to decrease (Figs 8 and 9b). We hypothesize that some parts of this maturing magma were ejected as late-stage spatter by the bursting of large, decoupled gas bubbles (Fig. 9c), which may have formed by two mechanisms: (1) As the dense lava that was ponded over the vent^35^ drained back down the conduit, it trapped air pockets. (2) Within the stagnant shallow conduit, continued coalescence led to the formation of large gas pockets. We envisage this process to be similar to the dynamic coalescence, gas bubble rise and subsequent spattering that was inferred to occur when magma was relatively stagnant beneath Pu’u‘Ō‘ō during the 2004–2005 activity^49^. Furthermore, the late-stage spatter is only found extremely proximal to the fissure (<10 m), on top of the solidified drained lava surface, suggesting that the energy released by gas/air pockets bursting was small. Lastly, the mismatch between the bulk vesicularity of the fissure drain-back lava and the late-stage spatter (Fig. 5) supports our interpretations about the parent magma/lava. If the late-stage spatter had formed entirely from the proximal draining lava, their bulk vesicularity would be the same; instead we observe that the late-stage spatter is much denser because material in the conduit (either stalled magma or recycled lava) had an extended opportunity for bubble rise and escape.

During the formation of the primary spatter deposits (northern ramparts and tree mould caps), spatter-fed rheomorphic lava flows moved south^33–35^. During lava flow advance we suggest that the vesicularity of the flow evolved via a combination of two processes (Fig. 9d). Firstly, as lava flows advance, gas bubbles rise to create a stratified flow with a gas rich upper zone directly beneath the quenched surface crust^50–52^. Secondly, when material is erupted on short timescales (seconds) the volatiles are not likely to be in equilibrium with the co-existing silicate melt; hence, during lava flow advance (minutes) volatiles diffuse out of the melt and cause bubble growth, thereby increasing the lava bulk vesicularity. Our field evidence (cross-cutting relationships; Section 2.2), indicates that, after well-established southward trending drainage channels had developed in the flow field, tectonic ground cracks opened or widened beneath the lava flow whilst it was still mobile (Fig. 2). We hypothesise that crack opening was accompanied by upward rushing hot gas from depth (similar to the April 1970 event) which disrupted the overlying mobile, draining lava flow and created secondary spatter (Fig. 9e). Also, during drainage into the cracks air pockets may have become trapped and subsequently burst (as described for the late-stage spatter). Either of these mechanisms, or both in combination, could distribute secondary spatter, in very small volumes, at vent-proximal locations, where the flow was most mobile (orange; Fig. 2c). Furthermore, the bulk vesicularity data (Fig. 5) suggests that the lava disruption preferentially mobilised the less dense, vesicular portion of the stratified lava to form secondary spatter with modal bulk vesicularity of 65 to 70%, similar to that of the primary spatter, but with a larger modal vesicle size. Finally, small amounts of lava continued to drain down the ground crack carrying most of the secondary spatter with it (Fig. 9f), only to leave small mounds on local topographic highs or en echelon jogs as observed in the field today (Fig. 1).

## Conclusions

In this study, we have identified and described the formation mechanisms of three different types of spatter associated with hawaiian low fountaining using field observations, bulk density measurements and micro-textural analysis. Using a N_Vm_ *versus* gas to melt volume ratio plot (Fig. 8), a framework has been set out to help classify different low fountaining products based on their micro-textural properties. Furthermore, we have shown that it is possible to find spatter deposits adjacent to ground cracks that did not act as magma conduits and purely act as sites of lava drain-back and disruption to form a secondary pyroclastic deposit. Field relationships and macro-scale observations (e.g. bulk density) were not enough alone to distinguish these deposits as different from primary fissure-fed pyroclastic deposits. Consequently, this may lead to vent and eruption misinterpretation, incorrectly informing hazards and probabilistic assessments, and spatter landforms within remotely sensed (planetary) data may require further investigation before their origin can be ascribed. However, through a micro-vesicularity study it has been shown that, based upon vesicle number density (N_Vm_), secondary deposits can be distinguished from the primary spatter generated by typical hawaiian fountaining. Therefore, N_Vm_ may be used as a classification metric to identity spatter types.

## Electronic supplementary material

Supplementary Information
