# Supplementary material for: Spatter matters – distinguishing primary (eruptive) and secondary (non-eruptive) spatter deposits
Source: Sci Rep. 2018 Jun 15;8:9179. doi: 10.1038/s41598-018-27065-1 (PMC6003959; doi:10.1038/s41598-018-27065-1)
Supplement: Supplementary file 1 — Supplementary Information [file 41598_2018_27065_MOESM1_ESM.pdf]

Supplementary Information (Tables S1 and S2)

to accompany:

Spatter matters – distinguishing primary (eruptive) and secondary (non-eruptive) spatter deposits

T.J. Jones[1*], B.F. Houghton[2], E.W. Llewellin[1], C.E. Parcheta[3] and L. Höltgen[1, 4]

[1] Department of Earth Sciences, Durham University, South Road, Durham, DH1 3LE, UK.

[2] Department of Geology & Geophysics, SOEST, University of Hawai‘i at Mānoa, Honolulu, HI 96822, USA.

[3] U.S. Geological Survey, Hawaiian Volcano Observatory, Hawaii Volcanoes National Park, HI, 96718, USA.

[4] Department of Earth and Environmental Sciences, LMU Munich, Theresienstr. 41, 80333 Munich, Germany.

Sample type	Clast name	Vesicularity [vol. %]	N_A[a] [#/cm^2]	N_V[b] [#/cm^3]	N_{Vm}[c] [#/cm^3]	V_g/V_m[d]	Median D[e] [mm]
Primary	8B	57.7	2.77E+3	1.05E+6	2.47E+6	1.37	0.74
Primary	8Ei	62.6	1.48E+3	4.96E+5	1.33E+6	1.69	0.61
Primary	4Q	59.7	1.23E+3	2.67E+5	6.61E+5	1.49	0.47
Primary	4A	52.3	1.83E+3	6.52E+5	1.37E+6	1.10	0.63
Primary*	1t C05	65.0	3.94E+3	8.90E+5	2.50E+6	1.88	0.50
Late-stage	1D q	27.1[f]	3.54E+2	7.59E+4	1.04E+5	0.37	1.07
Late-stage	5K	55.6	6.49E+2	1.44E+5	3.24E+5	1.27	0.78
Late-stage	6A q	32.3[f]	1.63E+3	4.99E+5	7.37E+5	0.48	0.50
Late-stage	1A q	31.9[f]	1.41E+2	9.64E+3	1.42E+4	0.47	0.91
Late-stage	1C q	53.7[f]	1.30E+3	3.50E+5	7.54E+5	1.16	1.36
Secondary	12H	49.0	9.56E+2	2.34E+5	4.58E+5	0.96	0.70
Secondary	12G	50.0	1.30E+3	3.72E+5	7.45E+5	1.00	0.49
Secondary	12Ni	73.4	5.70E+2	1.09E+5	4.10E+5	2.77	1.50
Secondary	12B q	43.3[f]	1.17E+3	4.73E+5	8.36E+5	0.77	0.71
Secondary	15Mii q	43.2[f]	1.14E+3	2.49E+5	4.38E+5	0.77	0.61

[a] Number of vesicles per unit area
[b] Number of vesicles per unit volume of clast matrix, volume of phenocrysts subtracted
[c] Number of vesicles per unit volume, referenced to melt only
[d] Ratio of gas to melt volume
[e] Median equivalent diameter, assuming a circular cross-section
[f] Sample comprises a quenched rim and interior, the quench image vesicularity is used/reported
*Tephra sample also described in Parcheta et al. (2013)

Table S1: Quantitative vesicle data from micro-textural analysis.

Spatter types compared	P-value
Primary – Late-stage	0.0053781
Secondary – Primary	0.0152794
Secondary – Late-stage	0.8311387

Table S2: ANOVA test results based on N_{Vm} distributed normally in linear space.
